# Age Differences in Elder Abuse Harm and Effective Services Offered by Adult Protective Services

**DOI:** 10.1093/geroni/igaa057.2320

**Published:** 2020-12-16

**Authors:** Pi-Ju Liu, Zachary Hass, Karen Conrad, Sara Stratton, Kendon Conrad

**Affiliations:** 1 Purdue University, West Lafayette, Indiana, United States; 2 University of Illinos at Chicago, Chicago, Illinois, United States; 3 San Francisco Adult Protective Services, San Francisco, California, United States; 4 University of Illinois at Chicago, Chicago, Illinois, United States

## Abstract

In this study, abuse, exploitation, and neglect (ANE) harm was measured by type of abuse using standardized items from the Identification, Services, and Outcomes (ISO) Matrix before Adult Protective Services (APS) interventions (pretest) and after APS interventions (posttest). Change scores from 1,472 older adults (average age 78-year-old; 57% female) and 591 younger adults (average age 53-year-old; 46% female) served by APS during the six months showed reduction of harm using posttest minus pretest. Nonetheless, older adult’s financial abuse harm (pretest=2.2, posttest=1.5) was higher than younger adults’ (pretest=1.5, posttest=1.2), while young adults scored higher in harm on all other types of abuse. Effective interventions differ by age group and by type of abuse, and will be discussed in detail. Results demonstrate the importance to consider vulnerable adult’s age and the etiology of abuse before implementing the services needed to effectively address ANE harm.

